# Comparison of single injection of intravitreal triamcinolone versus single injection of intravitreal bevacizumab in macular edema secondary to branch retinal vein occlusions with regard to changes in best corrected visual acuity and central macular thickness in the short term

**DOI:** 10.4103/0301-4738.49408

**Published:** 2009

**Authors:** Aditya S. Kelkar, Mehmood G. Sayyad, Jai A. Kelkar, Shreekant B. Kelkar, Reena Narvankar, Aarofil Shaikh

**Affiliations:** National Institute of Ophthalmology, Pune, India

Dear Editor,

We would like to present our experience of single injection of intravitreal triamcinolone (IVTA) versus single injection of intravitreal bevacizumab (IVB) in macular edema secondary to branch retinal vascular occlusions with regard to changes in best corrected visual acuity and central macular thickness in the short term.

Our retrospective study consisted of 18 patients (mean age 55.3 yers, 62% males) who received IVTA (0.1 ml containing 4 mg) or IVB (0.05 ml containing 1.25 mg) during the period October 2007 to February 2008. All the patients included had non-ischemic branch retinal vein occlusion with macular edema which was confirmed on fluorescein angiography. The patients having best corrected visual acuity (BCVA) of 20/40 or poor with persistent macular edema for at least three months were included. (median duration for IVTA 3.8 months; IVB 3.6 months and mean duration IVTA group: 3.5 months;; IVB group 3.1 months).

Visual acuity, intraocular pressure (IOP), anterior segment, posterior segment findings and macular thickness (performed on three-dimensional optical coherence tomography) were noted (pre-injection, post-injection Day 1, 7, 30, 90). Of 18 patients, eight received IVTA and the rest were given IVB. The BCVA was similar in both groups at first post-injection day (IVTA *P* = 0.470; IVB *P* = 0.100) i.e. majority of patients (six out of eight in the IVTA group and eight out of 10 in the IVB group) had stable visual acuity (no improvement) on first post-injection day. However, at seventh post-injection day (IVTA *P* = 0.024; IVB *P* = 0.640), one month (IVTA *P* = 0.043; IVB *P* = 0.244) and three months (IVTA *P* = 0.047; IVB *P* = 0.290) the BCVA was significantly better in the IVTA group. The percent reduction in central macular thickness was significant at one week, one month and three months (one week: IVTA 32.3% vs. Bevacizumab 24.9% [*P* = 0.029], one month: 36.2% vs. 21.4%[*P* = 0.014], three months: 39.8% vs. 25.4% [*P* = 0.014]). However, at first day post injection there was no significant difference in reduction of central macular thickness (20.36% vs. 20.45% *P* = 0.922).

At all follow-ups except first post-injection day, IOP was significantly higher in the IVTA group (one week: *P* = 0.021, one month: *P* = 0.034, three months: *P* = 0.029).

In a study by Pacolla *et al*.,[1] of 26 patients (26 eyes) with diabetic macular edema, the central macular thickness was significantly reduced in the IVTA group compared with the bevacizumab group at weeks 4, 8, 12 and 24 (*P* < 0.05).

BCVA was significantly higher at eight and 12 weeks in the IVTA group compared with the bevacizumab group (*P* < 0.05). On similar lines, our short-term results indicate that IVTA has better efficacy over bevacizumab in the management of macular edema secondary to branch retinal vein occlusions, specifically with regard to changes in BCVA and central macular thickness. However, the elevated IOP after IVTA is common[2] and since the prevalence of glaucoma/ocular hypertension is significantly higher in patients with central retinal vein occlusion and hemicentral retinal vein occlusion,[3] the IOP should be closely monitored in patients receiving IVTA.
